# Crinis Carbonisatus-Derived Carbon Dot Suspension Alleviates Temporal Lobe Epilepsy

**DOI:** 10.3390/ph18101481

**Published:** 2025-10-01

**Authors:** Yan Huang, Menghan Li, Liyang Dong, Chenxin He, Peng Zou, Minlong Xia, Bilin Jin, Siqi Wang, Zixuan Lu, Huihua Qu, Yue Zhang, Hui Kong

**Affiliations:** 1 School of Traditional Chinese Medicine, Beijing University of Chinese Medicine, Beijing 102401, China; 20230931031@bucm.edu.cn (Y.H.); lmh0698@163.com (M.L.); dly1613@163.com (L.D.); 20230931040@bucm.edu.cn (C.H.); zoupeng347@163.com (P.Z.); restat5pm@163.com (S.W.); luzixuan@163.com (Z.L.); 2 Center of Scientific Experiment, Beijing University of Chinese Medicine, Beijing 102401, China; quhuihuadr@163.com; 3 School of Life Sciences, Beijing University of Chinese Medicine, Beijing 102401, China

**Keywords:** Crinis Carbonisatus-derived carbon dots, suspension, temporal lobe epilepsy, MAPK, neuroinflammation, oxidative stress, excitotoxicity

## Abstract

**Background:** Temporal lobe epilepsy (TLE), a prevalent refractory focal epilepsy frequently complicated by comorbid anxiety and depression, poses significant therapeutic challenges due to the inadequate efficacy of current antiepileptic drugs in seizure control. Carbon dots (CDs) demonstrate notable biological activities and represent a promising class of nanomedicines for TLE intervention. **Methods:** This study established an eco-friendly calcination protocol to synthesize a novel suspension of Crinis Carbonisatus-derived carbon dots (CC-CDs) as a candidate therapeutic for TLE. **Results:** In a TLE mouse model, the CC-CDs suspension significantly inhibited phosphorylation of the MAPK pathway (p-JNK, p-ERK, p-p38; *p* < 0.01, *p* < 0.05), leading to reduced levels of pro-inflammatory cytokines (IL-6, IL-1β, TNF-α; *p* < 0.01, *p* < 0.05), upregulation of TGF-β1 (*p* < 0.01, *p* < 0.05), and restoration of antioxidant enzyme activities (SOD, GSH, CAT; *p* < 0.01, *p* < 0.05). These modifications subsequently regulated the Glu/GABA balance, alleviating excitotoxicity (*p* < 0.05), attenuating neuronal damage and Nissl body loss in hippocampal CA1/CA3 regions, and improving cognitive function alongside reducing anxiety-like behaviors (*p* < 0.01, *p* < 0.05). In vitro, the CC-CDs suspension suppressed LPS-induced apoptosis in BV2 cells. **Conclusions:** The CC-CDs suspension ameliorates TLE by inhibiting MAPK signaling, thereby reducing neuroinflammation and oxidative stress, rectifying Glu/GABA imbalance, attenuating excitotoxicity, and ultimately improving behavioral deficits. These findings underscore the therapeutic potential of CC-CDs suspension for TLE treatment.

## 1. Introduction

Epilepsy is a group of neurological disorders characterized by the occurrence of unpredictable and recurrent seizures, affecting approximately 70 million people worldwide [1,2]. Temporal lobe epilepsy (TLE) is the most common form of refractory focal epilepsy [3]. It typically originates from tissue damage in the hippocampus or amygdala, and its pathological basis is often closely associated with hippocampal sclerosis [4,5]. This condition not only triggers epileptic seizures but also frequently coexists with significant comorbidities, such as memory impairment, cognitive dysfunction, and anxiety-related emotional disorders [6].

However, current medications for TLE, primarily antiepileptic drugs, provide only symptomatic relief and fail to modify the underlying pathology or halt disease progression [7]. Furthermore, their use is hampered by significant challenges, including drug resistance and adverse reactions, complicating long-term disease management [8]. These limitations highlight the urgent need for innovative therapeutics targeting the core pathological mechanisms of TLE.

Recently, nanotechnology has brought innovations to the fields of medicine and pharmacology [9]. Carbon dots (CDs), a novel class of zero-dimensional carbon nanomaterials, offer distinct advantages for biomedical applications such as disease diagnosis, bioimaging, and biosensing due to their ultrasmall size (<10 nm), excellent water solubility, tunable photoluminescence, and biocompatibility [10,11,12]. Their diverse biological activities, including anti-inflammatory [13], antioxidant [14], antibacterial [15], antiviral [16], anti-allergic [17], anticancer [18], and immune-modulating effects [19], provide promising therapeutic strategies for clinical diseases.

Recent nano-based therapies for TLE have shown efficacy. KCaHNPs, ultrasmall catalytic nanoparticles, exert effects via mimicking superoxide dismutase, catalase, and other enzymes to neutralize reactive oxygen species, alleviate mitochondrial dysfunction, reduce neuronal apoptosis/ferroptosis, and inhibit glial activation, thus dampening neuroinflammation [8]. Fisetin-loaded chitosan nanoparticles, addresses the limitations of free fisetin (low solubility, rapid metabolism) by enhancing its pharmacodynamics. These nanoparticles ameliorate pilocarpine-induced TLE in mice through attenuating the ROS/TNF-α-NLRP3 inflammasome pathway, reducing seizures, and improving associated depression-like behavior and memory impairment [20]. However, both rely on exogenous drug loading or synthetic catalytic systems. Notably, despite the significant potential of CDs, the synthesis of many CDs usually involves chemical precursors and harmful reagents, which might limit their biomedical applications [21,22].

Numerous studies have confirmed the safety and high pharmacological activity of carbon dots derived from natural traditional Chinese medicines (TCM) [23,24]. TCM demonstrates therapeutic potential for epilepsy treatment [25]. Intriguingly, Chinese herbal medicines and their extracts can produce nano-components of charcoal drugs after high-temperature carbonization, and the process is similar to that of carbon dots [26]. Crinis Carbonisatus (Xue-yu-tan, CC), a classic TCM derived from carbonized human hair, has been widely used clinically for over two millennia. Classical Chinese medical books such as *Prescriptions for Fifty-two Diseases* describe its use for epilepsy and hemorrhage, and *Shennong Bencaojing* specifically records its efficacy in “treating infantile epilepsy and adult convulsions”, which directly corresponds to the core phenotypes of TLE. Our team previously isolated carbon dots from Crinis Carbonisatus (herein named Crinis Carbonisatus-derived carbon dots, CC-CDs) and validated their neuroprotective effects in ischemic stroke models [27]. Although these findings highlight their potential for neurological therapy, their efficacy against epilepsy remains unexplored. A critical challenge in the clinical translation of CC-CDs for antiepileptic therapy is nano-aggregation caused by size-dependent surface forces, which reduces active site accessibility and compromises colloidal stability [28]. To overcome this, we engineered a stabilized CC-CD suspension, ensuring colloidal stability to enhance bioavailability.

Recognizing that excitotoxicity, oxidative stress, and neuroinflammation are core pathological mechanisms in TLE [29], this study investigated the therapeutic epilepsy potential of a novel CC-CD suspension. Using a kainic acid (KA)-induced TLE mouse model and a lipopolysaccharide (LPS)-induced BV2 cell model, we systematically evaluated the anti-epileptic and neuroprotective effects in vivo, as well as the anti-inflammatory properties in vitro of CC-CD suspension. The findings provide strong preclinical evidence to support the translational research of CC-CD suspension as a novel intervention for TLE.

## 2. Results and Discussion

### 2.1. Characterization Findings of CC-CD Suspension

**Figure 1:**
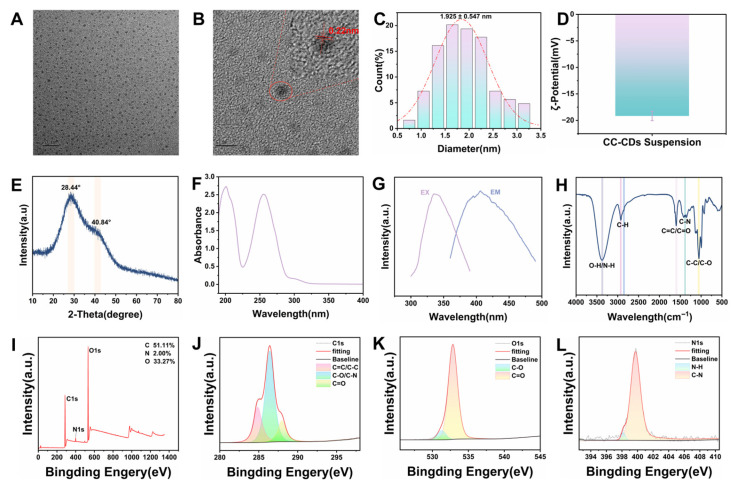
Characterization of CC-CD suspension. (**A**) Transmission electron microscope (TEM) image of CC-CD suspension. (**B**) High-resolution TEM (HRTEM) image of CC-CD suspension and lattice spacing (indicated by red arrows) of CC-CD suspension (d = 0.22 nm) (**C**) Histogram of particle size distribution. (**D**) The zeta potential of CC-CD suspension in DW. (**E**) X-ray Diffraction (XRD) pattern spectrum of CC-CD suspension. (**F**) Ultraviolet–Visible (UV-Vis) spectra of the CC-CD suspension. (**G**) Excitation and emission fluorescence spectra of CC-CD suspension. (**H**) Fourier Transform Infrared (FTIR) spectroscopy of CC-CD suspension. (**I**) Full X-ray Photoelectron Spectroscopy (XPS) spectrum of CC-CD suspension. (**J**) High-resolution XPS C1s spectrum. (**K**) High-resolution XPS O1s spectrum. (**L**) High-resolution XPS N1s spectrum.

Transmission electron microscope (TEM) analysis demonstrated that the CC-CD suspension exhibited uniform spherical nanostructures with particle sizes ranging from 0.5 to 3.5 nm with an average diameter of 1.925 ± 0.547 nm (Figure 1A–C). This ultra-small size facilitates biological barrier penetration [30]. High-resolution TEM (HRTEM) further revealed a lattice spacing of 0.22 nm (Figure 1B), corresponding to the (100) crystallographic planes of graphitic carbon, indicating short-range graphitic order in CC-CDs [31].

Zeta potential of CC-CD suspension in distilled water (DW) was −19.17 mV (Figure 1D), indicating good colloidal stability and favorable biocompatibility [32]. X-ray Diffraction (XRD) analysis showed broad peaks at 28° and 40° for CC-CDs (Figure 1E), corresponding to (002) and (100) planes of sp^2^-hybridized graphitic carbon [33,34,35]. The broad peaks can be attributed to the small particle size and the high degree of structural disorder in the CC-CDs, suggesting a lack of long-range crystalline order while confirming the presence of short-range graphitic features [36]. Collectively, the HRTEM and XRD results validate the presence of carbon dots with graphitic structures.

The optical properties of the CC-CD suspension were characterized using Ultraviolet–Visible (UV-Vis) and Fourier Transform Infrared (FTIR) spectroscopy. The UV-vis spectrum of the suspension (Figure 1F) exhibited absorption peaks at 200 nm and 255 nm, attributed to the π-π* transitions of the sp^2^-conjugated systems in aromatic rings containing C=C bonds [37,38]. Additionally, the Fluorescence (FL) spectrum. (Figure 1G) revealed a maximum excitation wavelength of 335 nm and a corresponding emission peak at 406 nm.

FTIR spectroscopy was used to analyze the chemical bonds and functional groups in the CC-CD suspension. In the FTIR spectra (Figure 1H), CC-CD suspension exhibited absorption peaks of –OH/–NH stretching vibration at 3388 cm^−1^, along with C-H stretching vibrations at 2928 cm^−1^ and 2848 cm^−1^ [39]. The absorption peak at 1602 cm^−1^ was ascribed to the stretching vibration of C=C/C=O [40]. The peak at 1384 cm^−1^ originated from the C-N stretching vibration. Moreover, the peak at 1054 cm^−1^ was assigned to C-C/C-O [41]. These functional groups confer water solubility and stability to the CDs [42,43].

X-ray Photoelectron Spectroscopy (XPS) further disclosed the element composition and allocation information of CC-CD suspension. The full XPS spectrum (Figure 1I) exhibited characteristic peaks at 286.16 eV (C1s), 399.89 eV (N1s), and 532.85 eV (O1s), indicating that the sample is primarily composed of carbon (51.11%), oxygen (33.27%), and nitrogen (2.00%). Deconvolution of the C1s spectrum (Figure 1J) revealed three components: 284.8 eV (C=C/C-C), 286.40 eV (C-O/C-N), and 287.97 eV (C=O). The O1s spectrum (Figure 1K) showed peaks at 531.28 eV (C=O) and 532.85 eV (C-O). The N1s spectrum (Figure 1L) displayed peaks at 399.73 eV (C-N) and 398.18 eV (N-H). This confirms surface functional groups (–COOH, –OH, –NH_2_) on CC-CDs in the suspension, consistent with FTIR data.

### 2.2. Cytotoxicity Detection

**Figure 2:**
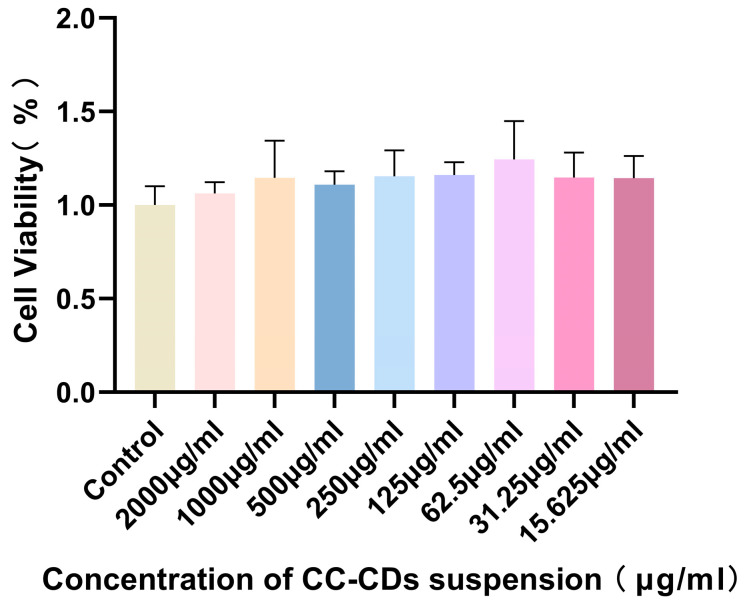
Effects of different concentrations of CC-CD suspension on BV2 cell viability. Data are expressed as mean ± SD (n = 6).

As shown in Figure 2, BV2 cell viability was assessed after treatment with CC-CD suspension at concentrations ranging from 15.625 to 2000 μg/mL. All tested concentrations maintained cell viability above 90% with no significant differences compared to the control group, indicating that CC-CD suspension exhibited excellent biosafety and no adverse effects on cell viability across the evaluated concentration range.

### 2.3. Seizure-Related Endpoint Detection

**Figure 3:**
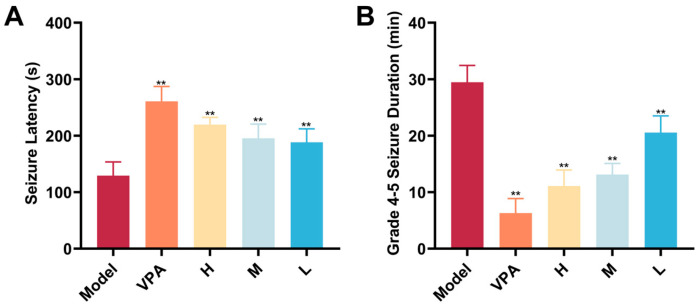
Effects of different treatments on seizure latency and grade 4–5 seizure duration in KA—induced TLE mice. (**A**) Seizure latency (s). (**B**) Total duration of grade 4–5 seizures within 2 h (min). Data are expressed as mean ± SD (n = 6). ** *p* < 0.01 versus Model group. Groups are defined as follows: Model (KA-induced TLE model group), VPA (valproic acid-treated group, positive control), H (high-dose CC-CD suspension group), M (medium-dose CC-CD suspension group), L (low-dose CC-CD suspension group).

Seizure severity and frequency are core indices for evaluating the therapeutic effect of interventions on temporal lobe epilepsy. As shown in Figure 3, compared with the Model group, both valproic acid (VPA) and CC-CD suspension at all doses significantly prolonged seizure latency and shortened severe seizure duration (*p* < 0.01).

The clinical significance of shortening the duration of severe seizures is substantial. Prolonged epileptic seizures can lead to multiple adverse consequences, including excessive neuronal excitation, excitotoxicity, and subsequent neuronal damage or even death, which in turn impairs brain functions such as cognition and memory [44]. The CC-CD suspension demonstrated the ability to effectively reduce seizure duration, indicating its potential to mitigate seizure-induced brain injury and to lower the risk of long-term complications such as cognitive impairment and psychiatric abnormalities.

### 2.4. Behavioral Assessment Findings

TLE is strongly associated with neuropsychiatric comorbidities including anxiety, depression, and cognitive dysfunction [45,46]. While conventional antiepileptics show limited efficacy against these comorbidities [47], our behavioral tests revealed CC-CD suspension’s therapeutic potential.

**Figure 4:**
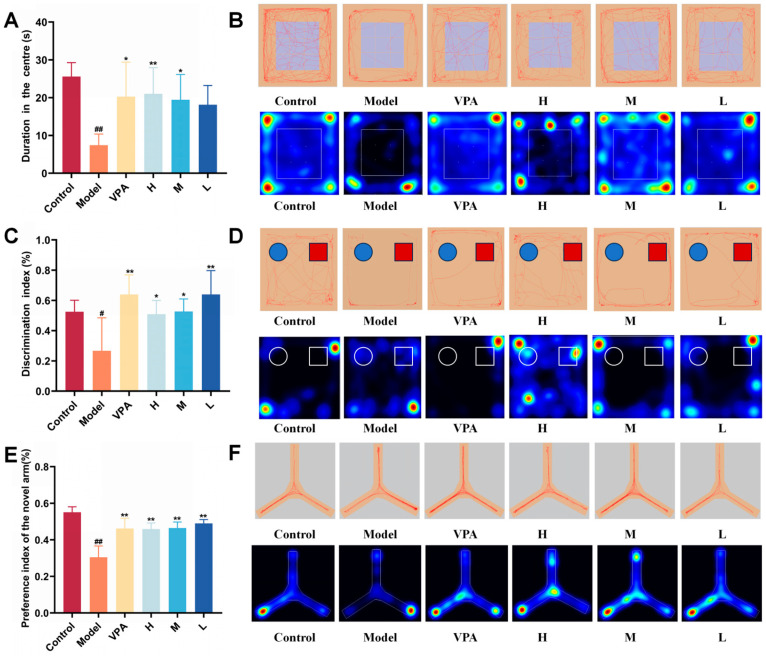
Behavioral observations of TLE mice. (**A**) Cumulative duration in the central zone of the Open Field Test (OFT). (**B**) Typical movement traces and heatmap of OFT. (**C**) Discrimination index of the Novel Object Recognition (NOR) test. (**D**) Typical movement traces and heatmap of NOR, where blue circles represent old objects and red squares represent new objects. (**E**) Preference index of the novel arm in the Y-Maze test. (**F**) Typical movement traces and heatmap of Y-Maze. Colors in heatmaps: Warmer colors (red, yellow) mean more movement; cooler colors (blue) mean less movement; black means almost no movement. Data are expressed as mean ± SD (n = 6). ^#^ *p* < 0.05, ^##^ *p* < 0.01 versus Control group; * *p* < 0.05, ** *p* < 0.01 versus Model group. Groups are defined as follows: Control (normal saline-treated group), Model (KA-induced TLE model group), VPA (valproic acid-treated group, positive control), H (high-dose CC-CDs suspension group), M (medium-dose CC-CDs suspension group), L (low-dose CC-CDs suspension group).

The Open Field Test (OFT) measured how active mice were and whether they showed anxiety-like behaviors in new environments [48]. TLE model mice spent significantly less time exploring the central zone compared to the control group (*p* < 0.01) (Figure 4A), indicating that epilepsy could cause anxiety-like behaviors in these mice. Compared with the model group, both VPA and the medium-dose CC-CD suspension significantly reversed this behavioral deficit (*p* < 0.05). Moreover, the high-dose CC-CD suspension demonstrated a superior improvement (*p* < 0.01). From typical trace diagrams and heatmap (Figure 4B), it was further revealed that the treated mice not only prolonged their dwelling time in the central zone but also showed more complex movement patterns. These findings confirm the therapeutic efficacy of CC-CD suspension in alleviating anxiety symptoms in TLE mice.

The Novel Object Recognition (NOR) test is a classic method for evaluating the learning and cognitive abilities of rodents [49]. Model group mice had a significantly lower novel object recognition index than the control group (*p* < 0.05) (Figure 4C), suggesting a deficit in learning and memory ability. Following intervention, both the high and medium doses of the CC-CD suspension notably increased this index (*p* < 0.05). Meanwhile, VPA and the low dose of the CC-CD suspension showed even more remarkable improvements (*p* < 0.01). From the typical trace diagrams and heatmap (Figure 4D), it was easy to observe that treatment group mice not only spent much more time in the novel object area but also increased the density of their exploration traces. This indicates that the CC-CD suspension can effectively improve the learning and memory functions of TLE mice by enhancing their exploratory behavior.

The Y-maze test (Y-Maze) is an important way to evaluate the spatial working memory ability of mice [50]. The preference index for the novel arm of the model group mice was significantly lower than that of the control group (*p* < 0.01) (Figure 4E). This confirms that TLE can cause damage to cognitive function. In contrast, both VPA and CC-CD suspension at different doses significantly increased the preference index for the novel arm in TLE mice (*p* < 0.01). By looking at the typical trace diagrams and heatmap (Figure 4F), it was clear that the treated mice went into the novel arm for exploration quite often. This shows that the CC-CD suspension can, to some degree, repair the impaired cognitive function of TLE mice.

### 2.5. Histological Evaluation of Brain Tissue

**Figure 5:**
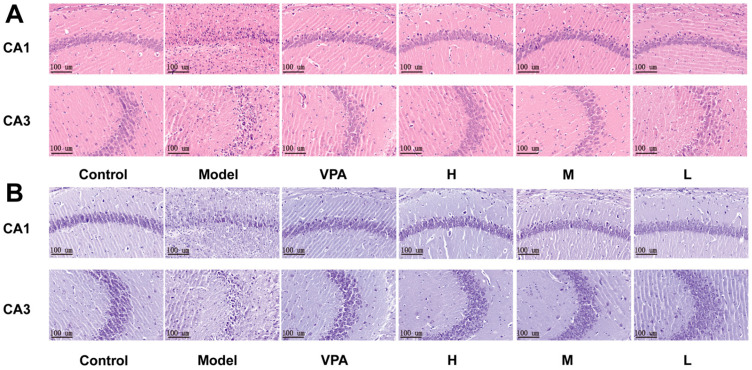
Histological evaluation of CC-CD suspension on TLE mouse brain tissue with H&E staining and Nissl staining. (**A**) H&E staining of hippocampal CA1/CA3 regions. (**B**) Nissl staining of hippocampal CA1/CA3 regions. Magnification 400×; Scale Bar 100 μm.

Hippocampal sclerosis represents a characteristic neuropathological finding in TLE patients [51], manifested by neuronal loss in the hippocampal CA1, CA3, and hippocampal hilar regions [52]. As shown by H&E staining (Figure 5A) and Nissl staining (Figure 5B), hippocampal CA1 and CA3 regions in control mice exhibited closely arranged neurons with intact morphology, round and centrally positioned nuclei containing clear nucleoli, homogeneous cytoplasmic staining, and abundant Nissl bodies. In contrast, the TLE model group exhibited disorganized neuronal alignment, triangular pyknotic nuclei, cytoplasmic shrinkage, abnormal nucleoplasmic ratios, blurred cytoplasmic boundaries with hyperchromasia, neuronal loss, and significantly reduced Nissl bodies, findings that confirmed TLE model establishment. Following intervention with VPA and CC-CD suspension at different doses, pathological damage was markedly alleviated: neuronal density increased with restored arrangement, Nissl bodies recovered significantly, and only sparse necrotic foci persisted, demonstrating neuroprotective effects.

### 2.6. The Effect of CC-CD Suspension on γ-Aminobutyric Acid (GABA) and Glutamate (Glu)

Cortical neuronal hyperexcitability in epilepsy arises from neuroinflammation-driven disruption of Glu/GABA homeostasis [53]. Pro-inflammatory cytokines like IL-1β and TNF-α directly potentiate NMDA receptor function, but also activate microglia, the brain’s resident immune cells [54,55,56]. Activated microglia release additional cytokines (including TNF-α) and excessive Glu, potentiating neuronal damage [56,57]. Furthermore, microglia-derived TNF-α stimulates astrocytes to increase Glu release, forming a self-reinforcing excitotoxic cycle [58,59]. This enhances NMDA receptor sensitivity, triggering Ca^2+^ overload and NOX2-mediated oxidative stress [60,61]. Concurrently, cytokines suppress GABA synthesis and receptor clustering, impairing GABAA receptor-dependent Cl^−^ influx that normally counteracts hyperexcitability [54,62]. This collectively drives a critical excitation-inhibition imbalance underlying seizures and comorbidities [63].

**Figure 6:**
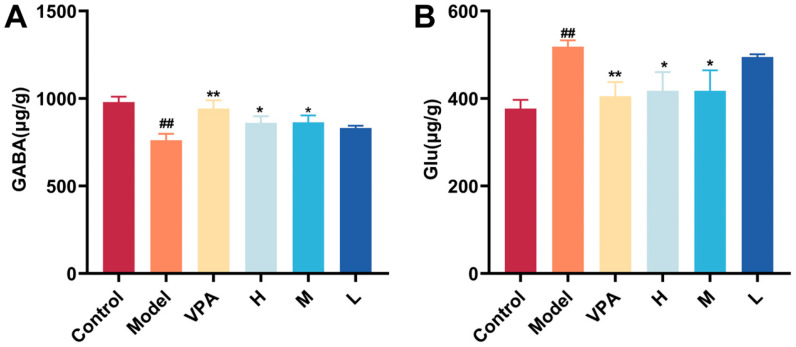
Effects of CC-CD suspension on GABA and Glu levels in TLE mouse brain tissue. (**A**) GABA level. (**B**) Glu level. Data are expressed as mean ± SD (n = 3). ^##^ *p* < 0.01 versus Control group; * *p* < 0.05, ** *p* < 0.01 versus Model group.

GABA and Glu levels in mouse brain tissue were quantified by High-Performance Liquid Chromatography (HPLC). As shown in Figure 6A,B, the Model group had significantly reduced GABA and elevated Glu levels compared to control (*p* < 0.01). Compared with the Model group, the VPA and high/medium-dose CC-CD suspension groups showed significantly increased GABA and decreased Glu levels (*p* < 0.01, *p* < 0.05). This restoration of GABA/Glu homeostasis parallels the effect of Anakinra, which suppresses cytokine-mediated NMDA hyperactivation [64]. The improvement indicates CC-CD suspension exerts beneficial effects, at least in part, by disrupting neuroinflammatory cascades and excitotoxic cycles, restoring excitatory/inhibitory balance.

### 2.7. The Effect of CC-CD Suspension on Oxidative Stress Levels in TLE Mice

Neuroinflammation and excitotoxicity trigger excessive ROS production, overwhelming the brain’s antioxidant defenses [65]. Redox homeostasis depends on balancing ROS production with antioxidant defenses, where SOD, GSH, and CAT act as first-line enzymes [66]. These enzymes neutralize ROS and maintain cellular redox balance [67,68]. Concurrently, oxidative stress amplifies neuronal excitotoxicity and drives pro-inflammatory cytokine release from activated glia [69]. This forms a bidirectional feedforward loop: neuroinflammation induces oxidative stress, which perpetuates neuroinflammation and disease progression.

**Figure 7:**
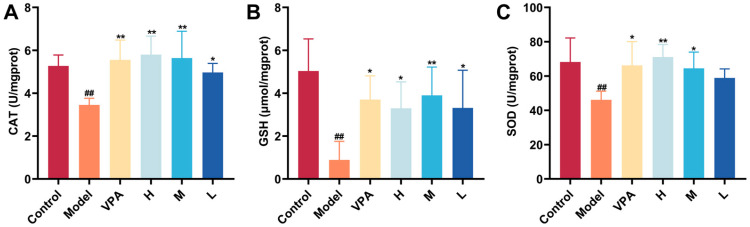
Effect of CC-CD suspension on oxidative stress indexes in TLE mouse brain tissue. (**A**) CAT activity. (**B**) SOD activity. (**C**) GSH level. Data are expressed as mean ± SD (n = 6). ^##^ *p* < 0.01 versus Control group; * *p* < 0.05, ** *p* < 0.01 versus Model group.

As shown in Figure 7A–C, in the KA-induced TLE model, the levels of SOD, GSH, and CAT in brain tissues were significantly depleted compared to the control group (*p* < 0.01), indicating antioxidant system collapse. Notably, In contrast to the model group, VPA and high/medium-dose CC-CD suspension increased CAT, SOD, and GSH levels in brain tissue (*p* < 0.01, *p* < 0.05), demonstrating restored redox homeostasis and antioxidant capacity.

### 2.8. The Effect of CC-CD Suspension on Inflammation Cytokine Levels in TLE Mice

The neuroinflammatory-oxidative milieu hinges on sustained microglia and astrocyte activation, central to epileptogenesis [70]. Activated glia release pro-inflammatory cytokines (IL-1β, TNF-α, IL-6), driving a self-sustaining loop of persistent glial hyperactivity and cytokine release [71]. This inflammatory environment further suppresses TGF-β1, a critical immunoregulatory cytokine responsible for anti-inflammatory and neuroprotective effects [72,73]. TGF-β1 downregulation in TLE impairs endogenous anti-inflammatory braking mechanisms, exacerbating pathology.

**Figure 8:**
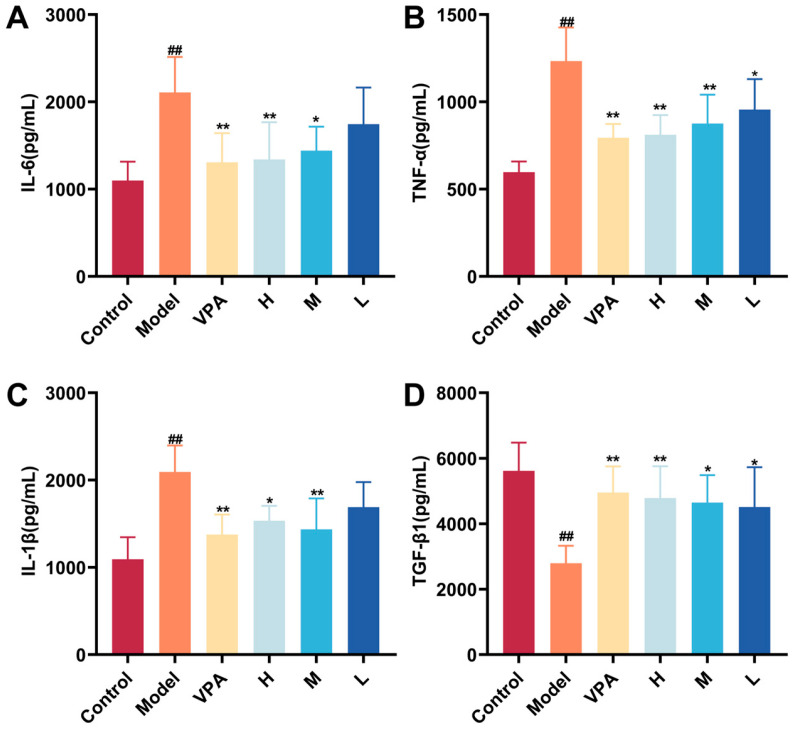
Effect of CC-CD suspension on inflammatory cytokines index in brain tissue of mice. (**A**) IL-6; (**B**) TNF-α; (**C**) IL-1β; (**D**) TGF-β1. Data are expressed as mean ± SD (n = 6). ^##^ *p* < 0.01 versus Control group; * *p* < 0.05, ** *p* < 0.01 versus Model group.

Figure 8A–D shows significantly higher pro-inflammatory cytokines (IL-1β, IL-6, TNF-α) and lower TGF-β1 in TLE model brain tissues compared to control (*p* < 0.01), reflecting glial hyperactivation and impaired neuroprotection. Importantly, CC-CD suspension and VPA reversed this: reducing IL-1β, TNF-α, IL-6 levels and restoring TGF-β1 in the model group, effectively dampening core neuroinflammation.

### 2.9. The Effect of CC-CD Suspension on MAPK Pathway-Related Protein Expression in TLE Mice

The dysregulated neuroinflammatory-redox milieu hyperactivates MAPK cascades (ERK, p38, JNK) [74,75,76]. ROS bursts and pro-inflammatory cytokines converge to activate these pathways [77]. Once activated, Phosphorylated MAPKs (p-ERK, p-p38, p-JNK) amplify upstream pathology via bidirectional crosstalk, driving inflammatory mediator production and oxidative damage. Pathologically, ERK hyperphosphorylation disrupts synaptic proteins, promoting hyperexcitability and destabilizing Glu/GABA balance [78,79]. Excessive JNK/p38 signaling drives inflammation and synergizes with ERK to exacerbate excitotoxicity [80,81]. This means overactive MAPK is a critical driver that amplifies the development of TLE.

**Figure 9:**
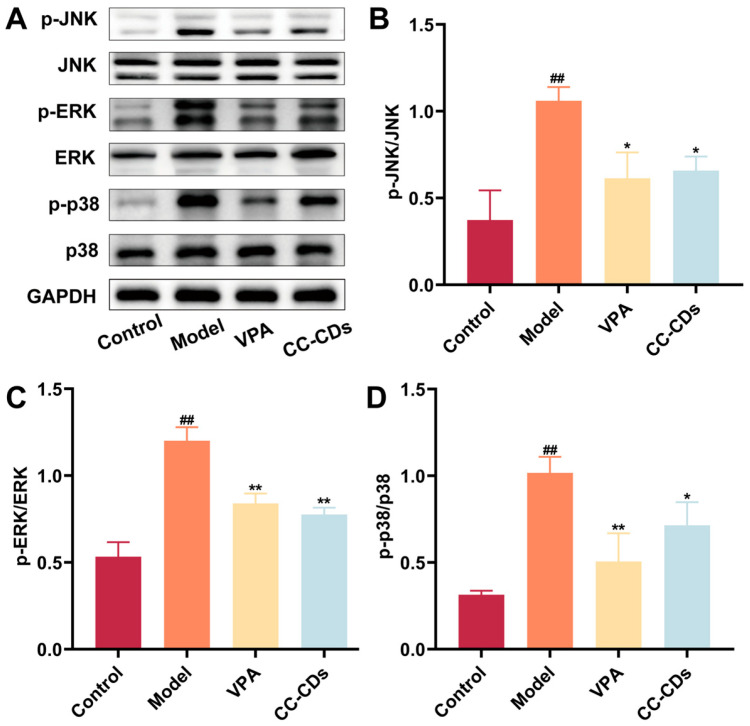
Effect of CC-CD suspension on MAPK pathway-related protein expression in TLE mouse brain tissue. (**A**) Western blot images of p-JNK, JNK, p-ERK, ERK, p-p38, p38, GAPDH. (**B**) Quantitative analysis of p-JNK/JNK ratio. (**C**) Quantitative analysis of p-ERK/ERK ratio. (**D**) Quantitative analysis of p-p38/p38 ratio. Data are expressed as mean ± SD (n = 3). ^##^ *p* < 0.01 versus Control group; * *p* < 0.05, ** *p* < 0.01 versus Model group.

Figure 9A–D shows elevated p-JNK/JNK, p-ERK/ERK, and p-p38/p38 ratios in KA-induced epileptic brains compared to the control group (*p* < 0.01), confirming MAPK activation in TLE. CC-CD suspension significantly suppressed these ratios (*p* < 0.05, *p* < 0.01), demonstrating its ability to modulate MAPK activity.

The suppression of MAPK hyperactivation by CC-CD suspension mechanistically links its anti-inflammatory, antioxidant, and neuroregulatory effects into a clearer cause-effect chain. By inhibiting MAPK signaling, CC-CD suspension attenuates neuroinflammation and reduces oxidative stress, which collectively restore the Glu/GABA balance. This correction of excitatory-inhibitory homeostasis subsequently mitigates neuronal hyperexcitability and seizure activity, ultimately leading to the amelioration of behavioral deficits in TLE.

### 2.10. Protective Effect of CC-CD Suspension on BV2 Cells

Microglia, as primary mediators of neuroinflammation, orchestrate inflammatory responses in CNS pathologies [82]. LPS activates microglia by binding to TLR4 receptors, triggering pro-inflammatory cytokine release and apoptosis, which exacerbates neuronal damage [83]. We evaluated CC-CD suspension’s neuroprotective effects in LPS-induced BV-2 microglial cells.

**Figure 10:**
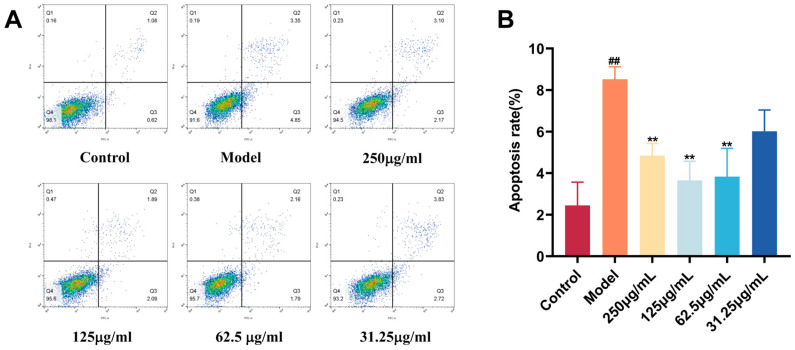
Effect of CC-CDs suspension on LPS-induced BV2 cell apoptosis. (**A**) Flow cytometry dot plots of BV2 cell apoptosis. In these dot plots, blue dots represent individual cells, and the green/yellow clustered area (typically in the Q4 region) represents viable cells. Dots in other quadrants indicate cells at different stages of apoptosis or necrosis. (**B**) Percentage of apoptotic BV2 cells. Data are expressed as mean ± SD (n = 3). ^##^ *p* < 0.01 versus Control group; ** *p* < 0.01 versus Model group.

Figure 10A,B shows significantly higher apoptosis in the LPS model group compared to the control group (*p* < 0.01). Pretreatment with CC-CD suspension at 250 μg/mL, 125 μg/mL, and 62.5 μg/mL significantly reduced BV2 cell apoptosis (*p* < 0.01), whereas the 31.25 μg/mL concentration showed no significant difference compared to the model group.

Apoptotic microglia not only lose their neuroprotective functions but also release pro-inflammatory mediators and cellular contents, triggering strong inflammatory cascades and exacerbating neuronal damage [84]. Suppressing microglial apoptosis mitigates neuroinflammation. Collectively, these results suggest CC-CD suspension maintains microglial survival under inflammatory stress, potentially disrupting microglia-driven pathological cascades.

### 2.11. Study Limitations

A notable limitation of this study is the relatively small sample size in in vivo experiments, which may reduce the statistical robustness of the results. This was primarily caused by the high mortality of experimental mice, variable modeling success of the KA-induced TLE model, and the insensitivity of some mice to KA. We will prioritize increasing the sample size in future studies to further validate the conclusions on CC-CD suspension’ therapeutic effects.

## 3. Materials and Methods

### 3.1. Materials

Human hair was obtained from the barbershop of Beijing University of Chinese Medicine (Beijing, China). VPA was acquired from Sigma-Aldrich Trading Co., Ltd. (Shanghai, China), and KA was purchased from MedChemexpress Biotechnology Inc., Princeton, NJ, USA. The dialysis membrane with a molecular weight cut-off of 1000 Da was procured from Beijing Ruida Henghui Technology Development Co., Ltd. (Beijing, China). Other general experimental materials were obtained from Sinopharm Chemical Reagents Beijing (Beijing, China). All experiments were conducted using DW.

Mouse TNF-α, IL-1β, IL-6, and TGF-β1 ELISA kits were provided by Shanghai Enzyme-linked Biotechnology Co., Ltd. (Shanghai, China). Mouse SOD, GSH, and CAT kits were purchased from Nanjing Jiancheng Bioengineering Institute (Nanjing, China). The BCA Protein Assay Kit was purchased from Shanghai Yamei Biomedical Technology Co., Ltd. (Shanghai, China). The cell counting kit-8 was procured from Wuhan Servicebio Technology Co., Ltd. (Wuhan, Hubei, China). The Annexin V-FITC Apoptosis Detection Kit was purchased from Biyuntian Biotechnology Co., Ltd. (Shanghai, China).

The following antibodies were used: Phospho-JNK (Tyr185) Polyclonal antibody, JNK Polyclonal antibody; Phospho-p38 MAPK (Thr180/Tyr182) Polyclonal antibody, p38 MAPK Polyclonal antibody; Phospho-ERK1/2 (Thr202/Tyr204) Polyclonal antibody, ERK1/2 Polyclonal antibody; GAPDH antibody, horseradish peroxidase (HRP)-conjugated Goat Anti-Rabbit IgG, and HRP-conjugated Goat Anti-Mouse IgG. All these antibodies were acquired from ProteinTech Group Co., Ltd. (Wuhan, Hubei, China).

### 3.2. Animals

SPF C57BL/6 male mice, weighing 20 ± 2 g, were purchased from Beijing SinoBest Biotechnology Co., Ltd. (Beijing, China) (Experimental Animal Quality Certificate No.: 11032241105181063). This study was conducted at the Experimental Animal Center of Beijing University of Chinese Medicine and was approved by the University’s Institutional Animal Care and Use Committee (BUCM-2024072203-3130). The environmental temperature was maintained at 24.0 ± 1.0 °C, the relative humidity was 55–65%, the light cycle was a 12 h day-night rhythm, and all animals had free access to food and sterile drinking water.

### 3.3. Preparation and Characterization of CC-CD Suspension

#### 3.3.1. Preparation of CC-CDs

Human Hair Pretreatment: Chemically untreated human hair (free of dyes, thermal damage, and impurities) was immersed in saturated NaHCO_3_ solution for 12 h, with the solution replaced once to remove oils and contaminants. The hair was heated in a 100 °C water bath for 1 h, followed by two 1 h washes with boiling DW to eliminate alkali residues. After drying at 60 °C for 12 h, the material was ready for calcination.

Calcination and Extraction: The dried hair underwent two-stage calcination in a muffle furnace: 70 °C for 20 min, then 350 °C for 1 h to produce carbonis crinis. The calcined product was pulverized and extracted twice with DW (1:30 g/mL, 100 °C, 1 h each). Combined extracts were filtered (coarse then fine), concentrated, and dialyzed against DW for ≥7 days (daily renewal until clear). The retentate was filtered through a 0.22 μm membrane, concentrated to 1 g/mL (carbonis crinis mass/volume), and stored at 4 °C.

#### 3.3.2. Formulation of CC-CD Suspension

5 mL of a 1 g/mL CC-CDs aqueous solution was added to a 15 mL centrifuge tube. The pre-swollen Sodium Carboxymethyl Cellulose stabilizer was introduced. Next, 0.05% mass fraction of nipagin ethyl (as a preservative) and 2.5% mass fraction of sucrose (as a flavoring agent) were weighed out and added to the tube, followed by thorough mixing. Finally, the solution was diluted to 10 mL with DW and ultrasonicated for 2 h to obtain the CC-CD suspension.

#### 3.3.3. Characterization of CC-CD Suspension

The particle size and morphology were characterized by TEM (Tecnai G2 20, FEI, Hillsboro, OR, USA). Atomic lattice spacing was determined using HRTEM (JEM-1230, JEOL, Tokyo, Japan). The Zeta potential was determined via electrophoretic light scattering (ELS; Zetasizer Nano ZS 90, Malvern Panalytical, Malvern, UK). Crystalline structure was analyzed by XRD (D8 Advance, Bruker, Billerica, Germany). UV-Vis absorption spectra and photoluminescence properties were recorded using the UV-Vis spectroscopy (CECIL 7200, Cambridge, UK) and FL spectrum (F-4500, Hitachi, Tokyo, Japan), respectively. Surface functional groups and elemental composition were characterized by FTIR spectroscopy (Nicolet iS50, Thermo Fisher, Fremont, CA, USA) and XPS (ESCALAB 250Xi, Thermo Fisher, Fremont, CA, USA).

### 3.4. Biocompatibility of CC-CD Suspension

The BV2 microglial cell line, sourced from Wuhan Pricella Biotechnology Co., Ltd. (Wuhan, Hubei, China), was cultured in BV2-specific medium. For viability assessment, cells were seeded in 96-well plates at a density of 1 × 10^5^ cells per milliliter with 100 microliters per well. After 24 h of adhesion, cells were exposed to CC-CD suspension at concentrations ranging from 15.625 to 2000 μg/mL. Following a 24 h incubation at 37 °C in 5% CO_2_, cells were washed with phosphate-buffered saline. Viability was assessed using the CCK-8, and absorbance at 450 nm was measured after a 2 h incubation period.

### 3.5. TLE Model Establishment and Drug Administration

Sixty male C57BL/6 mice were acclimated for 3 days and then divided into two groups using a random number table method: control group (n = 8), model group (n = 52). The randomization was performed by an independent researcher not involved in subsequent experiments, ensuring no significant differences in baseline body weight and general condition between the two groups before modeling. The model group received an optimized dose of 22 mg/kg KA to establish TLE, while the control group was administered 0.9% saline [85,86,87]. Behavioral manifestations were continuously monitored for 2 h post-modeling. According to the modified Racine scale (Table 1) [88], mice exhibiting stage 4 seizures (with ≥5 stage 4 or higher seizures within 30 min after the first stage 4 episode, lasting ≥ 30 min) were defined as achieving status epilepticus, indicating successful modeling. Non-sensitive responders failing to meet these criteria were excluded.

Forty surviving mice with status epilepticus were further randomized into five groups (n = 8 each) via a random number table by an independent researcher, balancing seizure severity and body weight: model, VPA, and CC-CD suspension high-dose (H), medium-dose (M), and low-dose groups (L). After 1 day recovery period, all groups received daily intragastric administration of 0.2 mL for 14 days, with the following contents: the control and model groups were given DW; the VPA group received 20 mg/mL VPA solution; and the CC-CDs groups were administered suspensions at concentrations of 0.12 mg/mL (H), 0.08 mg/mL (M), and 0.04 mg/mL (L), respectively. Half an hour after the last drug administration, KA (22 mg/kg) was re-injected to simulate kindling status [89]. We recorded two key seizure-related endpoints for each group: latency to the first epileptic seizure and total duration of grade 4–5 severe seizures within 2 h. All recordings and analyses of these endpoints were conducted by two independent researchers blinded to group assignments; any discrepancies were resolved via joint review against the modified Racine scale to eliminate observer bias.

### 3.6. Behavioral Observations

To evaluate therapeutic efficacy, behavioral tests (open field, novel object recognition, Y-maze) were conducted after 14 days of treatment, and a 24 h rest period following the KA repeated injection and seizure recording, ordered from least to most invasive to minimize stress interference. Mice were acclimated to the testing environment for 60 min prior to experiments. All apparatuses were cleaned with 75% ethanol between trials to eliminate odor cues. The tests were performed by researchers blinded to group assignments, with data recording and analysis also conducted under blind conditions to avoid subjective bias. Equipments were provided by the Scientific Research Center of Beijing University of Chinese Medicine.

#### 3.6.1. Open Field Test

Locomotor activity and anxiety-like behavior were assessed in a dimly lit arena (50 × 50 × 30 cm). Mice were placed in the center and allowed to explore freely for 5 min. Movement trajectories were analyzed using EthoVision XT7.

#### 3.6.2. Novel Object Recognition

Cognitive function was assessed using a two-phase novel object recognition test in a black square chamber (50 × 50 × 30 cm). During the habituation phase, mice freely explored two identical objects for 5 min. After a 2 h interval, one object was replaced with a novel prism-shaped object, and exploration times for the novel (Tnovel) and familiar (Told) objects were recorded over 5 min. The discrimination index (DI), reflecting recognition memory, was calculated as: DI = Tnovel/(Tnovel + Told) × 100%.

#### 3.6.3. Y Maze Test

Spatial working memory was evaluated using a Y-maze apparatus with three arms (50 × 10 × 20 cm): Start Arm (SA), Novel Arm (NA), and Other Arm (OA). In the habituation phase, NA was blocked, and mice explored SA and OA for 10 min. After 1 h, NA was unblocked, and mice freely explored all arms for 5 min. The number of entries into each arm (NNA, NSA, NOA) was recorded. The novel arm preference index, indicating spatial memory retention, was calculated as: NNA/(NNA + NSA + NOA) × 100%.

### 3.7. Histopathological Analysis

Mouse brain tissue samples were fixed in 4% paraformaldehyde solution, subsequently embedded in paraffin, and sectioned into 4 μm thick slices. Hematoxylin–eosin (HE) and Nissl staining were performed on the paraffin-embedded sections. The stained sections were examined under a light microscope to evaluate neuronal morphology in the hippocampal region and the integrity of Nissl bodies. Morphological assessment of the stained sections was conducted by two researchers blinded to group assignments, with consensus results adopted as final data.

### 3.8. Detection of Brain Tissue Neurotransmitters

Frozen brain tissues were weighed, homogenized, and extracted with 1 mL of 50% aqueous methanol by shaking for 1 h. The homogenate was centrifuged at 12,000 rpm for 10 min at 4 °C. The supernatant was collected and mixed with internal standard and isopropanol containing 0.1% formic acid. Samples were derivatized using AccQ-Tag reagent, heated at 55 °C for 10 min, diluted, and analyzed by HPLC (UPLC I-Class, Waters Corporation, Milford, MA, USA) for qualitative and quantitative detection of GABA and Glu. Target amino acid concentrations were calculated using standard curves.

### 3.9. Enzyme Linked Immunosorbent Assay

The brain tissue was homogenized in normal saline to prepare 10% brain homogenate, which was further centrifuged at 4000 rpm for 10 min at 4 °C to obtain supernatant. The levels of inflammatory cytokines including TNF-α, IL-1β, IL-6, TGF-β1 in brain tissue were determined using commercial ELISA kits. In addition, the oxidative stress indicators including SOD, GSH, and CAT levels were measured and all operations were performed according to the manufacturer’s instructions.

### 3.10. Western Blot

Proteins were extracted from mouse brain tissue, and their concentrations were quantified using the BCA assay. After normalization to a uniform concentration, the proteins were separated by sodium dodecyl sulfate-polyacrylamide gel electrophoresis. The resolved proteins were transferred onto polyvinylidene fluoride membranes, which were then incubated overnight at 4 °C with primary antibodies. Following three washes with TBST, the membranes were incubated with HRP-conjugated secondary antibodies for 1 h at room temperature. Protein bands were visualized via chemiluminescence and quantified using ImageJ software (1.54p). GAPDH expression was used as an internal loading control.

### 3.11. Flow Cytometry

BV2 cells were seeded in 6-well plates at a density of 5 × 10^5^ cells per milliliter with 2.5 mL per well. Cells were divided into control, LPS-induced model, and CC-CDs-treated groups with concentrations ranging from 31.25 to 250 μg/mL, each group containing three replicates. After 2 h of pretreatment with CC-CDs-containing medium, LPS-containing medium was added for 24 h induction. Cells were collected, digested using EDTA-free trypsin, stained with Annexin V-FITC and propidium iodide, and analyzed by flow cytometry with a BD FACSCalibur instrument. Apoptosis rates were quantified using FlowJo V10 software.

### 3.12. Statistical Analysis

Experimental data were expressed as mean ± standard deviation ($\bar{x}$ ± *s*). Statistical analysis was performed using GraphPad Prism 9.0 software. The normality of the data was evaluated by the Shapiro–Wilk test, with a *p* > 0.05 indicating a normal distribution. For data with normal distribution and homogeneous variances, one-way analysis of variance (ANOVA) was used, followed by the Tukey post hoc test for pairwise comparisons. For data with non-normal distribution or unequal variances, the Kruskal–Wallis non-parametric test was applied. Statistical significance was defined as *p* < 0.05. Statistical significance was defined as *p* < 0.05.

## 4. Conclusions

CC-CD suspension demonstrated good biocompatibility and conferred protective effects on BV2 microglial cells. In KA-induced TLE mice, it significantly suppressed seizures, improved cognitive function and anxiety-like behaviors, and alleviated hippocampal neuronal damage. The therapeutic effects may be mediated, at least in part, through the inhibition of the MAPK signaling pathway, which appears to subsequently alleviate neuroinflammation and oxidative stress, contribute to the restoration of the Glu/GABA balance, and ultimately ameliorate excitotoxicity and behavioral deficits. Thus, CC-CDs represent a promising comprehensive therapeutic strategy for TLE.

## Figures and Tables

**Table 1:** Racine Scoring Criteria.

Grade	Corresponding Behavioral Characteristics
0 Grade	Normal activity, no stress response.
1 Grade	Facial muscle twitching, blinking, chewing, salivation, etc., reduced activity.
2 Grade	Rhythmic nodding or wet—dog shakes visible.
3 Grade	Unilateral forelimb twitching and spasm visible, no hindlimb spasm, upright posture.
4 Grade	Bilateral forelimb twitching, spasm, falling, and hindlimbs upright.
5 Grade	Generalized tonic spasm, forced standing or falling, death.

## Data Availability

The original contributions presented in this study are included in the article. Further inquiries can be directed to the corresponding authors.

## Abbreviations

The following abbreviations are used in this manuscript: TLE, Temporal lobe epilepsy; CDs, Carbon dots; CC-CDs, Crinis Carbonisatus-derived carbon dots; KA, kainic acid; LPS, lipopolysaccharide; TCM, Traditional Chinese medicine; VPA, Valproic acid; DW, deionized water; TEM, transmission electron microscopy; HRTEM, high-resolution TEM; XRD, X-ray Diffraction; FL, Fluorescence; UV-Vis, Ultraviolet–visible; FTIR, Fourier transform infrared; XPS, X-ray photoelectron spectroscopy; OFT, Open Field Test; NOR, Novel Object Recognition; Y-maze test, Y-Maze;HE, Hematoxylin–eosin; HPLC, High-Performance Liquid Chromatography; GABA, γ-aminobutyric acid; Glu, glutamate; DI, discrimination index; SA, Start Arm; NA, Novel Arm; OA, Other Arm.
